# Interleukin-34, a Novel Paracrine/Autocrine Factor in Mouse Testis, and Its Possible Role in the Development of Spermatogonial Cells In Vitro

**DOI:** 10.3390/ijms21218143

**Published:** 2020-10-30

**Authors:** Alaa Sawaied, Eitan Lunenfeld, Mahmoud Huleihel

**Affiliations:** 1 The Shraga Segal Department of Microbiology, Immunology, and Genetics, Faculty of Health Sciences, Ben-Gurion University of the Negev, Beer Sheva 84105, Israel; alaasa@post.bgu.ac.il; 2 The Center of Advanced Research and Education in Reproduction (CARER), Faculty of Health Sciences, Ben-Gurion University of the Negev, Beer Sheva 84105, Israel; lunenfld@bgu.ac.il; 3 Department of OB/GYN, Soroka Medical Center, Beer Sheva 8410501, Israel

**Keywords:** testis, spermatogenesis, in vitro culture of spermatogonial cells, interleukin-34, autocrine/paracrine factors, colony-stimulating factor-1 (CSF-1)

## Abstract

Spermatogenesis is the process of spermatogonial stem cell (SSC) proliferation and differentiation to generate sperm. This process is regulated by cell–cell interactions between Sertoli cells and developing SSCs by autocrine/paracrine and endocrine factors. It is also affected by cells in the interstitial compartment, such as Leydig cells and peritubular cells. Here, we demonstrate, for the first time, the presence of interleukin-34 (IL-34) in Leydig, Sertoli, and peritubular cells and in the premeiotic, meiotic, and postmeiotic cells. Its receptor, colony-stimulating factor-1 (CSF-1), has already been demonstrated in Leydig, Sertoli, premeiotic, and meiotic cells. IL-34 was detected in testicular homogenates and Sertoli cell-conditioned media, and was affected by mouse age. We showed that the addition of IL-34 in vitro to isolated cells from the seminiferous tubules of 7-day-old mice, using the methylcellulose culture system (MCS), increased the percentages and expression of the premeiotic cells (VASA), the meiotic cells (BOULE), and the meiotic/postmeiotic cells (ACROSIN) after four weeks of culture, when examined by immunofluorescence staining (IF) and qPCR analysis. It is possible to suggest that IL-34 is a novel paracrine/autocrine factor involved in the development of spermatogenesis. This factor may be used in future therapeutic strategies for the treatment of male infertility.

## 1. Introduction

Spermatogenesis is a complicated process that includes proliferation and differentiation of spermatogonial stem cells (SSCs) to generate sperm [1,2,3,4,5,6]. This process starts from SSCs that are located near the basement membrane of the seminiferous tubules, which undergo several mitotic divisions followed by meiotic divisions and spermiogenesis to form elongated spermatid [1,2,3,4,5,6]. Cells of each stage express specific markers, such as VASA, PLZF, and SALL4 for the premeiotic stage, BOULE, CREM-1, and ACROSIN for the meiotic stage, and ACROSIN and PROTAMINE for the postmeiotic stage [3,4,7,8,9,10,11]. This process is under the regulation of endocrine and testicular autocrine/paracrine factors [1,4,5]. Testicular somatic cells such as Leydig cells, peritubular cells, and Sertoli cells produce various autocrine/paracrine factors that are involved in the regulation of spermatogenesis such as colony-stimulating factor-1 (CSF-1), stem cell factor (SCF), glial cell line-derived neurotrophic factor (GDNF), and others [1,2,3,4,12,13,14,15]. Receptors for these factors were demonstrated on spermatogonial cells [12,16,17,18].

Interleukin-34 (IL-34) is a protein that is produced mainly by neurons and keratinocytes [19,20]. It is also secreted by a subset of small cells in the spleen, lymph nodes, kidney tubes, and testicles [21]. IL-34 is an essential cytokine for the proliferation, differentiation, and survival of macrophages and Langerhans cells [20,22]. Studies have shown that IL-34 promotes the development, survival, and function of microglia and Langerhans cells in vivo, and stimulates the viability of monocytes [22]. IL-34 binds and acts through two receptors: IL-34 receptor and CSF-1R [20,23,24]. IL-34 receptor is a protein-tyrosine phosphatase [20,25]. Knockout (KO) mice, lacking the IL-34 gene, are fertile, and have no renal dysfunction and no defects in monocytes, macrophages, or dendritic cells [21].

The CSF-1 receptor was demonstrated on spermatogonial stem cells, and was shown to induce the proliferation of spermatogonial cells [15,18].

In previous studies of our group, we were able to induce proliferation and maturation of spermatogonial cells in vitro using three-dimensional in vitro culture systems such as the soft agar culture system (SACS) and the methylcellulose culture system (MCS) [3,26,27,28,29]. Using these systems, we were able to induce mouse and human spermatogonial cells to differentiate to postmeiotic stages, including sperm-like cells [26,28,29].

The aims of the present study were to examine the presence of IL-34 in testicular cells and its levels during sexual maturation, in addition to its possible role in the spermatogenesis process.

In the present study, we demonstrated, for the first time, the presence of IL-34 in mouse testicular somatic cells (Sertoli, Leydig, and peritubular cells) and in premeiotic, meiotic, and postmeiotic cells. In addition, we showed that IL-34 could induce the proliferation and maturation of mouse spermatogonial cells to meiotic/postmeiotic stages in vitro using MCS.

## 2. Results

### 2.1. Localization and Expression Levels of IL-34 in Testicular Tissue

**Figure 1:**
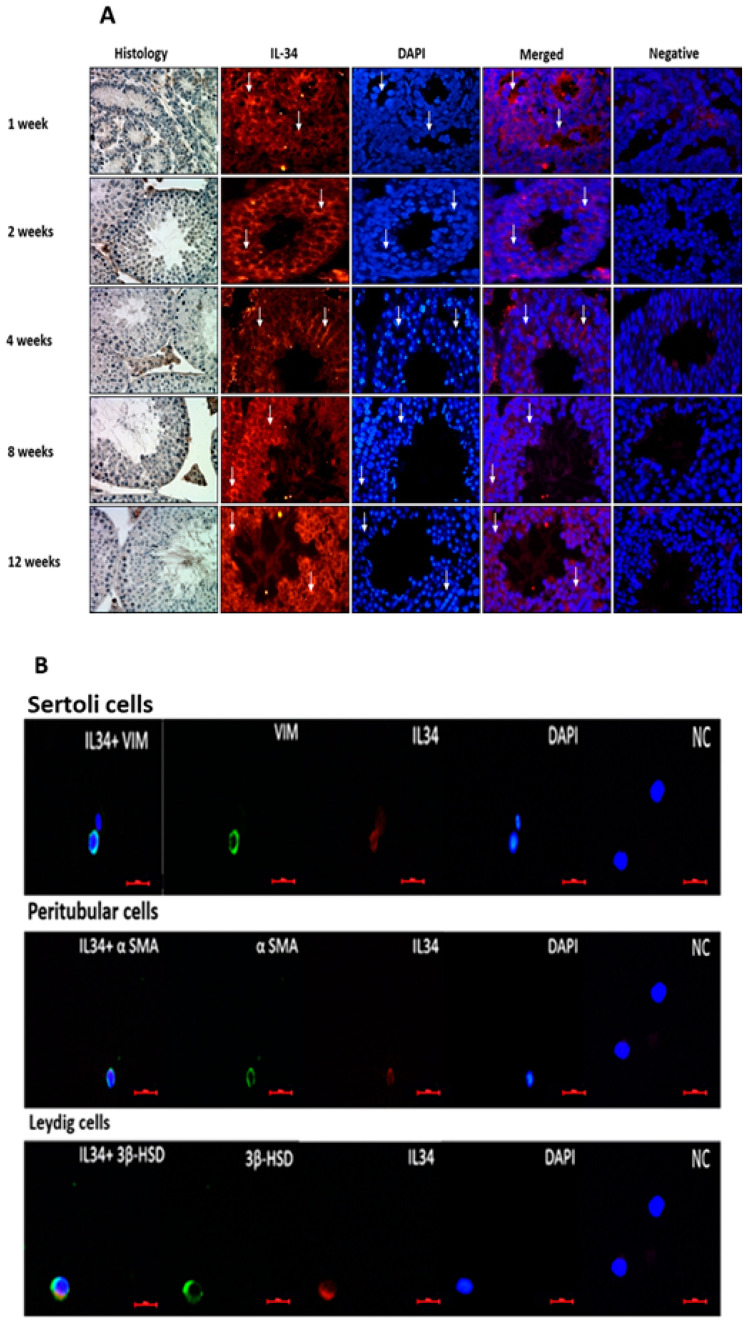

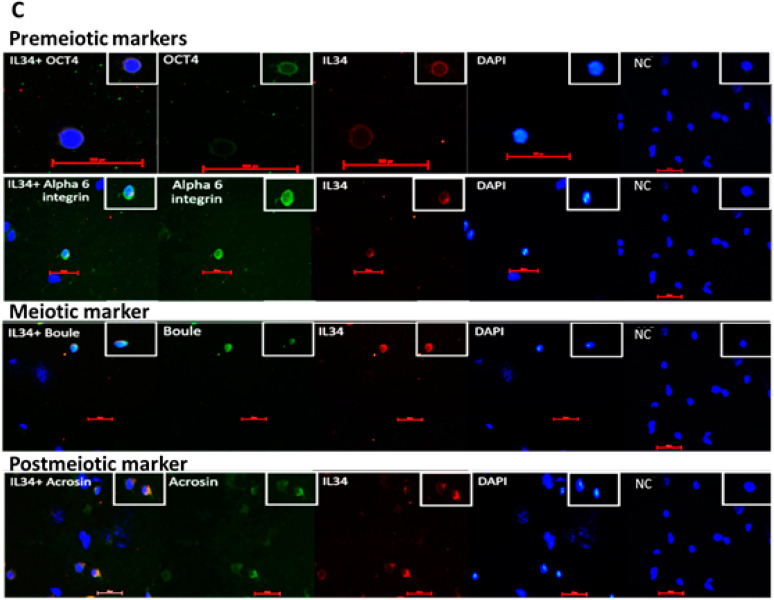
Cellular localization of interleukin-34 (IL-34) in mice testicular cells. Testicular histology (Histology) shows the structure and cellular types of the interstitial compartment and the seminiferous tubules. The cellular localization of IL-34 in mouse testicular tubular cells from different ages (1, 2, 4, 8, 12 weeks) (arrows show stained cells, scale bar: 10 μm) was examined by immunofluorescence staining (IF) using specific primary antibodies and Cy3 with the relevant secondary antibodies (IL-34; red staining) (400) (**A**). IL-34 was localized in the branches of Sertoli cells (clearly seen in the seminiferous tubules of 4-week-old mice). DAPI was also used to show the nucleus of the cells (DAPI; blue staining). In addition, IL-34 was localized in isolated Sertoli cells, peritubular cells, and Leydig cells as examined by double IF staining of IL-34 (Cy3, red color) and vimentin (a Sertoli cell marker), a-SMA (a peritubular cell marker), and 3HSD (a Leydig cell marker), respectively (green color) (**B**). IL-34 (red color) was localized in isolated testicular premeiotic cells (using OCT4 and a-6-integrin as specific markers, meiotic cells (BOULE was used as a specific marker), and postmeiotic cells (ACROSIN was used as a specific marker (Dylight 488, green) (**C**). DAPI was also used to show the nucleus of the cells (DAPI; blue staining). Sections with merge of IL-34 (red color), cell markers (green color), and DAPI are presented (Merge). As a negative control (NC), we stained tissues or cells as described in the Materials and Methods section. The negative controls for cells in Figure 1B are similar (the same picture) since we used the same secondary antibodies for their staining in the same experiment. Additionally, the negative controls for cells in Figure 1C are similar (the same picture) since we used the same secondary antibodies for their staining in the same experiment. Scale bar: 10 μm.

Testicular tissues from different ages of mice (1 w, 2 w, 4 w, 8 w, 12 w) (see also histology of the sections from these ages; left side of Figure 1) were stained with a specific antibody to IL-34 by immunofluorescence staining (IF). Our results demonstrated that IL-34 is present in different cells in the seminiferous tubules and in the interstitial compartment along all examined ages (Figure 1A).

Using double IF staining for isolated testicular somatic cells (Sertoli, peritubular, and Leydig cells), from 6-week-old mice and IL-34, we demonstrated that all the examined cells were positively stained to IL-34 (Figure 1B). Moreover, using double IF staining for premeiotic cells (OCT4 and a-6-integrin), meiotic cells (BOULE), and postmeiotic cells (ACROSIN) and IL-34, we showed positive staining for IL-34 in all the examined premeiotic, meiotic, and postmeiotic cells (Figure 1C).

### 2.2. Effect of Age on the Levels of IL-34 in Testicular Homogenates and Sertoli Cell-Conditioned Media

**Figure 2:**
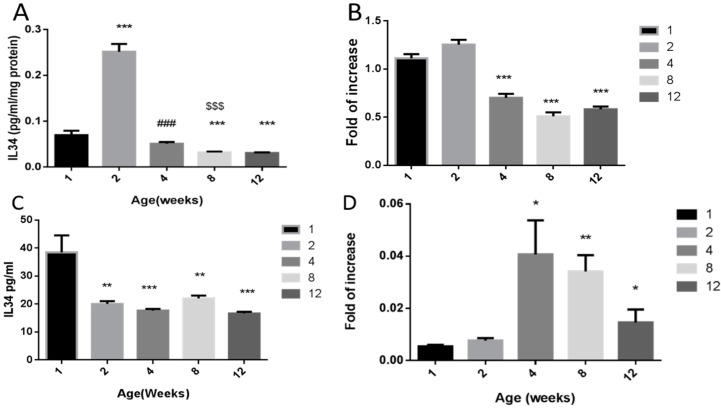
Effect of age on IL-34 levels in testicular homogenates and Sertoli cell cultures. The protein levels of IL-34 were examined in testicular homogenates (presented as pg/mL/mg protein) (**A**) and conditioned media of Sertoli cell cultures (presented as pg/mL) (**C**) isolated from mice of different ages (1, 2, 4, 8, 12 weeks) using specific ELISA as described in the Materials and Methods section. The mRNA expression levels of IL-34 in the examined testicular tissues (**B**) and Sertoli cell cultures (**D**) were evaluated by real-time PCR analysis using specific primers. *, compared to 1w. #, compared to 2w. $, compared to 4w. * *p* < 0.05, ** *p* < 0.01, and *** *p* < 0.001. ^###^ *p* < 0.001. ^$$$^ *p* < 0.001. *n* = 6 (number of mice for each time point; Figure 2A,B). For Sertoli cell cultures, we performed 3 independent experiments with 3–5 repeats in each experiment for the same age. For each age, we used 6 mice.

Our results showed different protein levels in mouse testicular homogenates at different ages (1 w, 2 w, 4 w, 8 w, 12 w), as examined by specific ELISA. They were significantly increased in 2-week-old mice compared to 1-week-old mice, and significantly decreased to similar levels of 1-week-old mice in the other examined ages (4–12 weeks old) (Figure 2A). However, the expression levels of IL-34 levels in mouse testicular homogenates of different ages (1 w, 2 w, 4 w, 8 w, 12 w), as examined by qPCR analysis, were significantly higher in 1- and 2-week-old mice compared to other examined ages (4–12 weeks old) (Figure 2B).

The protein levels of IL-34 in conditioned media of Sertoli cell cultures, which were isolated from 1-week-old mice, were substantially higher compared to those in conditioned media from Sertoli cell cultures isolated from 2-week-old to 12-week-old mice (Figure 2C). However, the RNA expression levels of IL-34 were significantly higher in Sertoli cell cultures isolated from 4-week-old to 12-week-old mice compared to 1-week-old and 2-week-old mice (Figure 2D). It should be noted that there was a significant decrease in the expression levels of IL-34 in Sertoli cell cultures isolated from 12-week-old mice compared to 4-week-old mice (Figure 2D).

### 2.3. Localization of CSF-1R in Testicular Cells

**Figure 3:**
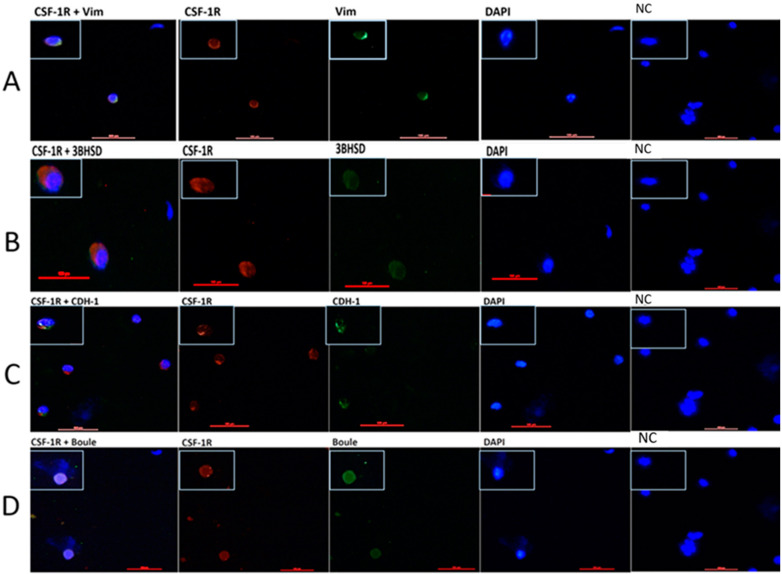
Localization of CSF-1R in Sertoli, Leydig, premeiotic, and meiotic cells. The colocalization of CSF-1R was examined in isolated Sertoli cells (**A**) and Leydig cells (**B**) using specific markers as mentioned in Figure 1. The localization of CSF-1R was examined in the premeiotic cells (CDH1 was used as a specific marker) (**C**) and meiotic cells (BOULE was used as the specific marker) (**D**) by double IF staining of CSF-1R (Cy3, red color) and the antibodies specific to each cell type (Dylight 488, green). Cells with merge of CSF-1R (red color), cell markers (green color), and DAPI are presented (Merge). As a negative control (NC; the same picture), we stained cells in the same secondary antibodies (double staining) in the same experiment, as described in the Materials and Methods section. Scale bar: 10 μm.

Our results showed that CSF-1R is present in Sertoli and Leydig cells when examined by double IF staining using specific antibodies to each marker (Figure 3A,B). In addition, we showed that CSF-1R is present in CDH1 cells (a marker of premeiotic spermatogonial cells) and BOULE cells (a marker of meiotic cells) by double IF staining using specific antibodies to each cell marker (Figure 3C,D).

### 2.4. Involvement of IL-34 in the Development of Spermatogenesis In Vitro

**Figure 4:**
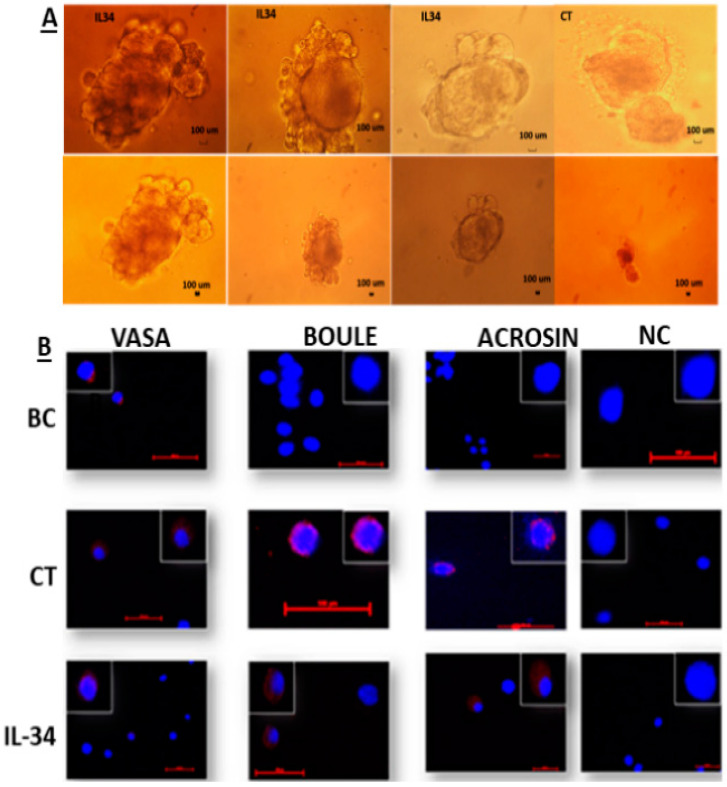
Development of spermatogonial cells in vitro in methylcellulose culture system. Cells were isolated from the seminiferous tubules of 7-day-old mice, by enzymatic digestion. These cells were cultured in a methylcellulose culture system (MCS) in the absence or presence of IL-34 (1–10,000 pg/mL). After 4 weeks of culture, the developed colonies and cells (**A**) were collected, and the cells were fixed and stained by IF staining, using specific antibodies for markers of the premeiotic (VASA), meiotic (BOULE, and ACROSIN), and postmeiotic (ACROSIN) cells (**B**). BC, before culture; CT, cells collected from cultures in the absence of IL-34; IL-34, cells collected from cultures treated with IL-34.

Our results show the development of clusters or colonies from isolated seminiferous tubule cells of 7-day-old mice, after 4 weeks of culture in vitro using the methylcellulose culture system. These developed clusters or colonies were found in the presence and absence of IL-34 (Figure 4A). We did not identify a significant difference in the size and/or number of the developed colonies in the presence or absence of IL-34. We also did not recognize any negative effect on the viability of the cells when we added high concentrations of IL-34 (1000 and 10,000 pg/mL).

Staining of cells from the developed cultures showed the presence of markers for premeiotic (VASA), meiotic (BOULE), and meiotic/postmeiotic (ACROSIN) cells (in control culture without IL-34; CT and IL-34-treated cultures; IL-34), whereas cells before culture (BC) were stained only for VASA cells, but not for BOULE or ACROSIN (Figure 4B).

### 2.5. Effect of IL-34 on the Levels of Developed Premeiotic, Meiotic, and Postmeiotic Cells In Vitro

**Figure 5:**
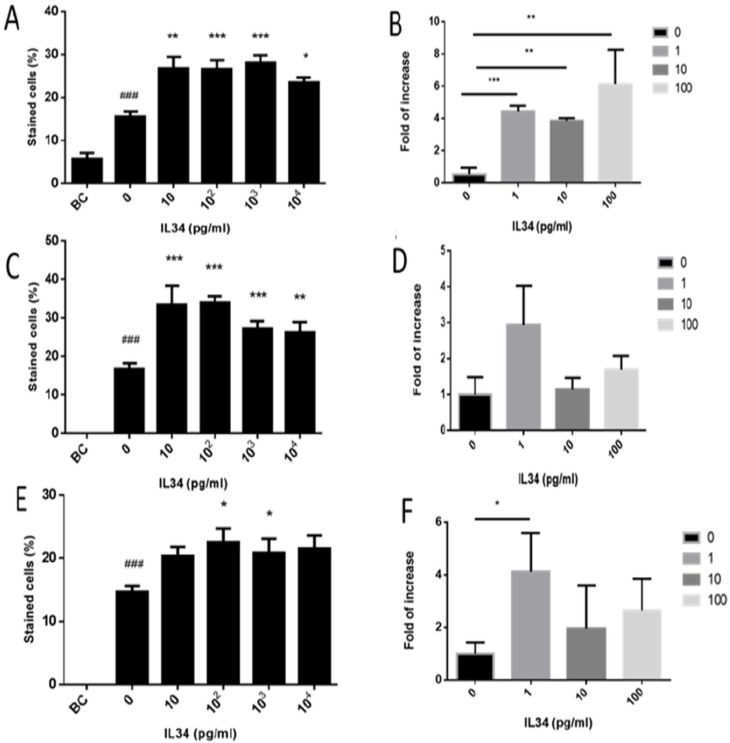
Effect of IL-34 on the development of spermatogonial cells in vitro in methylcellulose culture system. Isolated cells from the seminiferous tubules were cultured in vitro in the absence (0) or presence of different concentrations of IL-34 (1–10,000 pg/mL) as described in Figure 4. The percentages and expression levels of VASA (**A**,**B**, respectively), BOULE (**C**,**D**, respectively), and ACROSIN-positive cells (**E**,**F**, respectively) before culture (BC), after 4 weeks of culture in the absence of IL-34 (0), or in the presence of IL-34 (1–10,000 pg/mL) are presented. The percentages of cells were determined according to specific IF staining of each cell type from all cells in the examined field, and the RNA expression was determined by qPCR analysis using specific primers for each marker. *, compared to control. #, compared to BC. We performed 3 independent experiments with 3–5 repeats for each treatment in each experiment. For each experiment, we used 10 mice. * *p* < 0.05, ** *p* < 0.01, and *** *p* < 0.001. ^###^ *p* < 0.001.

Our results show that in vitro culture of spermatogonial cells for 4 weeks in MCS significantly induced the percentages of the premeiotic cells (VASA-positive cells) in the culture (control; CT) compared to before culture (BC). The addition of different concentrations of IL-34 (10 pg/mL to 10,000 pg/mL) considerably increased the percentages of the premeiotic cells (VASA) (Figure 5A) and their expression levels (Figure 5B) in vitro compared to CT, in a dose-dependent manner. It should be noted that the optimal effect of IL-34 on the percentages of VASA was at 10 pg/mL, whereas on the expression levels, it was at 100 pg/mL.

Furthermore, our results indicate that in vitro culture of spermatogonial cells for 4 weeks in MCS significantly induced the percentages of the meiotic cells (BOULE-positive cells) in the culture (CT) compared to those before culture (BC) in which meiotic cells (BOULE-positive cells) could not be detected (Figure 5C). The addition of different concentrations of IL-34 (10 pg/mL to 10,000 pg/mL) significantly increased the percentages (Figure 5C) and the expression levels (Figure 5D) of the BOULE cells in vitro compared to those in CT, in a dose-dependent manner. It should be noted that the optimal effect of IL-34 on the percentages of BOULE-positive cells was at 10 pg/mL, whereas on the expression levels, it was at 1 pg/mL.

Our results show that in vitro culture of spermatogonial cells for 4 weeks in MCS significantly induced the percentages of the meiotic/postmeiotic cells (ACROSIN-positive cells) in the culture (CT) compared to those before culture (BC) in which they could not be detected. The addition of different concentrations of IL-34 (10 pg/mL to 10,000 pg/mL) considerably increased the percentages (Figure 5E) and the expression levels (Figure 5F) of the ACROSIN cells in vitro compared to those in CT, in a dose-dependent manner. It should be noted that the optimal effect of IL-34 on the percentages of ACROSIN was at 100 pg/mL, whereas on the expression levels, it was at 1 pg/mL.

### 2.6. Effect of IL-34 on the Expression Levels of Growth Factors in Cells Developed in MCS

**Figure 6:**
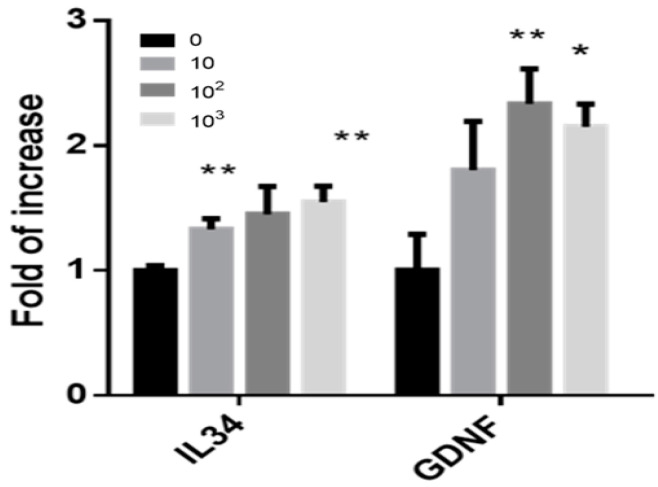
Effect of IL-34 on the expression levels of IL-34 and GDNF in cells developed in vitro in methylcellulose culture system. Isolated cells from the seminiferous tubules were cultured in vitro in the absence (0) or presence of different concentrations of IL-34 (10, 100, 1000 pg/mL) as described in Figure 4. Cells and colonies or clusters were collected after 4 weeks of culture and the RNA expression levels of IL-34 and GDNF were determined by qPCR analysis using specific primers for each factor. *, compared to control (0). We performed 3 independent experiments with 3–5 repeats for each treatment in each experiment. For each experiment, we used 10 mice. * *p* < 0.05, ** *p* < 0.01.

The addition of IL-34 to isolated cells from the seminiferous tubules of 7-day-old mice led to the development of clusters or colonies after 4 weeks in the in vitro culture in MCS (Figure 4A). Extraction of RNA from those cultures showed that the addition of IL-34 significantly increased the expression levels of IL-34 and GDNF in the developed cells, in a dose-dependent manner as determined by qPCR analysis (Figure 6).

## 3. Discussion

Our results show for the first time the presence and expression of the cytokine IL-34 in testicular cells, including the somatic cells (Sertoli, peritubular, and Leydig cells), and also testicular germ cells from the premeiotic (VASA, a-6-integrin), meiotic (BOULE, ACROSIN), and postmeiotic (ACROSIN) stages. We also showed for the first time the presence of CSF-1R, which is considered to be the receptor for IL-34 in the somatic, Sertoli, Leydig, premeiotic (CDH1), and meiotic cells (BOULE). According to these results, IL-34 could be considered as a testicular autocrine/paracrine factor that may be involved in the regulation of testicular functions and spermatogenesis. It should be noted that, in previous studies using transcriptome and proteomic database, IL-34 protein was not identified in isolated spermatogonial cells at different stages of differentiation [30]. This contradictory result may be related to a different methodology used and to their sensitivities.

Our results show that high levels of IL-34 protein were detected in testicular homogenates of 2-week-old mice compared to 1-week-old and 4–12-week-old mice, while on the RNA expression levels, testicular homogenates from 1-week-old and 2-week-old mice were similar and significantly higher compared to 4-week-old to 12-week-old mice. This discrepancy between protein levels and RNA expression in the 1-week-old and 2-week-old mice could be related to transcription–translation regulation. However, the significant decrease in IL-34 expression and protein levels compared to 2-week-old mice could be linked to an increase in the levels of the gonadotropins and testosterone at these ages. These results may suggest that testicular IL-34 is under the regulation of hormones involved in the development of spermatogenesis. It is also possible that the differences of IL-34 levels between ages could be related to differences in the type of spermatogenic cells present in the testis and/or the stage of development and functionality of the somatic cells (Sertoli and Leydig cells) at the examined ages.

Our results showed that conditioned media from cultures of Sertoli cells isolated from 1-week-old mice produced significantly high levels of IL-34 compared to other ages (2-week-old to 12-week-old mice). However, the expression levels of IL-34 were considerably higher in conditioned media of Sertoli cells isolated from 4-week-old to 12-week-old mice compared to 1-week-old and 2-week-old mice. These results may indicate that the regulation of IL-34 protein production and its RNA expression levels differs in Sertoli cells. It is possible that this regulation could be related to the differentiation of Sertoli cells from embryonic to adult age (higher differentiation after 2 weeks old).

The effect of age on the protein and RNA expression levels of IL-34 differed in testicular homogenates and cultures of Sertoli cells, which indicates that IL-34 in the homogenates originates and represents different testicular cells, which responded to age differentiation from only Sertoli cells. Moreover, it is possible to infer that high levels of hormones (mainly FSH and testosterone) (4–12-week-old mice) may be involved in the downregulation of RNA expression of IL-34 in the testis, and in their absence (in Sertoli cell cultures), the expression levels of IL-34 might increase. This effect could also be related to the presence or absence of different types of spermatogonial cells at the different stages of differentiation at the examined ages.

Previous studies have shown low RNA expression levels in the testis from adult mice compared to other organs in the body such as cerebrum, pituitary, salivary gland, ear, epidermis, and others [31,32,33]. In the present study, we showed that IL-34 RNA expression and protein levels are higher in immature ages compared to adult mice using qPCR analysis and specific ELISA for protein levels.

Even though we identified IL-34 in different cell types of testicular cells, which may suggest a physiological role for IL-34 in the development of spermatogenesis and male fertility, the IL-34 KO mice are fertile [21]. This may indicate that IL-34 is not an obligatory or critical factor for the development of normal spermatogenesis, and that other factor(s) in the testis may overlap its role in this process.

In order to evaluate the possible role of IL-34 in the spermatogenic process, we added various concentrations of recombinant IL-34 (IL-34) to isolated cells from seminiferous tubules of 7-day-old mice, and cultured in vitro using MCS. The MCS was demonstrated in our laboratory as a reliable system to induce the proliferation (development of colonies or clusters of cells) and maturation of spermatogonial cells from mice and humans to meiotic and postmeiotic stages including, in some cases, generation of sperm-like cells [27,28,29,34].

The addition of various concentrations of IL-34 to isolated cells from seminiferous tubules of 7-day-old mice in MCS for 4 weeks did not show a remarkable effect on the size and/or number of the developed colonies and on the viability of the cells. However, it significantly increased the development of premeiotic cells (VAS), meiotic cells (BOULE), and meiotic/postmeiotic cells (ACROSIN) compared to the control even in the low concentrations used (1 and 10 pg/mL). The high concentrations of IL-34 (100–10,000 pg/mL) did not show a negative effect on the viability of the cells but showed similar results to the low concentrations (1, 10 pg/mL) on the induction of cells from different stages of spermatogenesis. This may suggest that the optimal effect of IL-34, at least under in vitro conditions, is in the low concentrations. These results suggest the involvement of testicular IL-34 in the regulation of the different stages of spermatogenesis. It should be noted that, in the control conditions (without addition of IL-34; 0 pg/mL), the spermatogonial cells proliferated in vitro. This could be as a result of production of paracrine factors from the cells in the culture in the absence of IL-34. However, in the presence of IL-34 their proliferation was increased. It is possible that IL-34 affects spermatogonial behavior (proliferation/differentiation) directly (they express CSF-1 receptor) and/or indirectly through its effect on somatic cells (which express CSF-1 receptor) present in the culture (in response, these cells produce factors that may induce the proliferation/differentiation of the spermatogonial cells). Again, this effect may be different under in vitro conditions compared to in vivo.

Furthermore, the addition of IL-34 to the MCS system significantly increased the expression levels of IL-34 and GDNF in the developed cells. Thus, it is possible to suggest that IL-34 can act as an autocrine/paracrine regulatory factor that is involved in the development of spermatogenesis, both in vivo and in vitro. This suggestion is supported by our study results. We demonstrated the presence of both IL-34 and its receptor (CSF-1R) in the same cells, that is, somatic cells and different type of testicular germ cells that are involved in the development of spermatogenesis. The presence of IL-34 receptor on the spermatatogenic cells has not yet been demonstrated. Therefore, the possibility that IL-34 may affect spermatogenesis, also through the IL-34 receptor, cannot be excluded.

Previous studies have demonstrated the presence of CSF-1R on thy-1 spermatogonia and indicated that CSF-1 has an effect on the proliferation of specific types of SSCs [16]. We demonstrated the presence of CSF-1R on other spermatogonial cells such as CDH1 and even in meiotic cells such as BOULE. These results may suggest that different types of SSCs or spermatogonial cells could be directly affected by CSF-1 and IL-34. In addition, the presence of CSF-1R on Leydig and Sertoli cells may signify an additional mechanism of action of CSF-1 and IL-34 in the regulation, proliferation, and differentiation of SSCs and/or spermatogonial cells and meiotic cells. This could be indirectly through factors produced by these somatic cells. Our results showed the effect of IL-34 on the proliferation and differentiation of spermatogonial cells in vitro even in the presence of FCS or knockout serum replacement (KSR) and other growth factors such as GDNF, leukemia inhibitory factor (LIF), fibroblast growth factor (FGF) and epidermal growth factor (EGF) (unpublished), which are produced mainly by Sertoli cells. These results may suggest IL-34 as being a novel paracrine/autocrine testicular factor that might be involved in the development of spermatogenesis. However, it is not probably a limiting factor for this process since IL-34 knockout mice are fertile [21].

In summary, we showed that IL-34 and its receptor (CSF-1R) are produced by testicular somatic cells and germ cells at different stages of differentiation. It is suggested that the levels of IL-34 could be under hormonal regulation. IL-34 induced spermatogonial cell proliferation and differentiation in vitro to meiotic/postmeiotic cells. Thus, IL-34 could be considered as a novel testicular autocrine/paracrine factor that is involved in the regulation of spermatogenesis under normal conditions. Furthermore, this factor may be used in future therapeutic strategies (such as in vitro maturation of spermatogonial cells) for the treatment of male infertility.

## 4. Materials and Methods

### 4.1. Animals

Our study was confirmed by the Ben-Gurion University Ethics Committee for Animal Use in Research (No. IL-16-04-2018). Hsd:ICR (CD-1^®^) (ICR; Institute of Cancer Research) mice (Harlan Laboratories, Jerusalem, Israel) at different ages (1–12 weeks old) were used.

### 4.2. Chemicals and Reagents

Roswell Park Memorial Institute Medium (RPMI)-1640 media, penicillin, streptomycin, and fetal calf serum (FCS) were purchased from Beit HaEmek Biological Industries (Beit HaEmek, Israel). Collagenase IV (from clostridium histolyticum) and DNAase were obtained from Sigma-Aldrich (St. Louis, MO, USA). The levels of IL-34 protein were determined by enzyme-linked immunoassay (ELISA) (Mouse IL34-439107, BioLegend, San Diego, CA, USA). The range of the standard curve was 2–2000 pg/mL; sensitivity was 8.4 ± 1.8 pg/mL.

### 4.3. Isolation and Culture of Mouse Sertoli Cells

Sertoli cell cultures were performed according to Huleihel et al., 2013 [33] with some modifications. Briefly, Sertoli cells were enzymatically isolated from seminiferous tubules of 1–12-week-old ICR mice. After 4 days of incubation, Sertoli cell cultures were washed and hypotonically shocked to remove residual germ cells, followed by the addition of new minimum essential media (MEM) media containing 5% FCS. After 24–48 h of incubation at 5% CO_2_ and 37 °C, cells were washed and fresh MEM containing 5% FCS was added for an additional 24 h. Conditioned media were collected and used to evaluate the levels of IL-34 protein by ELISA, and cells were used to extract RNA to evaluate the expression levels of IL-34 by qPCR analysis. Some of the isolated seminiferous tubule cells were used to examine the presence of IL-34 or CSF-1R in somatic cells and germ cells using double immunofluorescence staining.

### 4.4. Immunofluorescence Staining of Mouse Testicular Tissue

Immunofluorescence staining of 4µm thick sections from formalin-fixed, paraffin-embedded testicular tissue blocks was performed as described by Huleihel et al., 2013 [33]. Polyclonal rabbit anti-mouse IL-34 antibodies (USBI, United States Biological-141047, Salem, MA, USA) (0.2 mg/mL; 1:40; final dilution) or polyclonal rabbit anti-mouse CSF-1R antibodies (LS-C164350, LifeSpan BioSciences, Seattle, WA, USA) (1:200 final dilution) were used as primary antibodies. The fluorescence antibody (Cy-3; donkey anti-rabbit antibodies; 1:1000; Jackson ImmunoResearch, West Grove, PA, USA) were applied as secondary antibodies. After overnight incubation at 4 °C, the antibodies were washed by PBS and DAPI staining and Molecular Probes (Santa Cruz Biotechnology, Inc., Santa Cruz, CA, USA) were added. Negative controls were included for each specimen using PBS/casein/FCS/BSA or relevant IgG isotype instead of the primary antibodies.

### 4.5. Immunofluorescence Staining of Cells Isolated from Seminiferous Tubules

Double immunofluorescence staining for IL-34 or CSF-1R with Sertoli cell marker (vimentin) (Sc-7557, Santa Cruz Biotechnology, Inc., 1:50 final dilution), Leydig cell marker (3β-hydroxysteroid dehydrogenase; 3βHSD) (Sc-30820, Santa Cruz Biotechnology, 1:100 final dilution), peritubular cell marker (alpha smooth muscle actin; αSMA) (Ab21027, Abcam, Cambridge, UK), premeiotic cells (VASA, NBP224558, Novus Biologicals, Centennial, CO, USA;1:50 final dilution), α-6-integrin, (Sc-6596, Santa Cruz Biotechnology, 1:40), meiotic cells (BOULE) (LS-B7334, LifeSpan BioSciences, 1:120 final dilution), and postmeiotic cells (ACROSIN) (NBP2-14260, Novus Biologicals, 1:2000 final dilution) was performed to identify the cellular origin and compartment of IL-34 or CSF-1R as described [33,34].

The immunostaining was performed according to our previous study [33], using the above mentioned primary antibodies: polyclonal goat anti-mouse vimentin (1:50) or polyclonal rabbit anti-mouse IL-34 (0.2 mg/mL) followed by the relevant secondary antibodies: Fluorescein-conjugated antibodies (Cy3, donkey anti-rabbit, 1:1000 dilution (Jackson ImmunoResearch) and Dylight 488, rabbit anti-goat antibodies, 1:100 dilution (KPL, Milford, MA, USA) were used for visualization of the signal according to the suppliers’ directions. After 1 h of incubation, the slides were washed in PBS and subsequently subjected to DAPI staining (Santa Cruz Biotechnology). Negative controls were included for each specimen using PBS containing FCS/BSA/relevant IgG isotype instead of the primary antibodies. Double staining of CSF-1R- (LS-C164350, LifeSpan BioSciences; 1:200 final dilution) or vimentin-positive cells was performed in isolated tubular cells from 6-week-old mice as described [33].

Slides were examined for staining using the Nikon eclipse 50i microscope (Tokyo, Japan).

### 4.6. Preparation of Testicular Homogenates

Testicular homogenates prepared from 1- to 12-week-old mice were performed as described by Huleihel et al., 2013 [33]. Protein IL-34 levels in testicular homogenates and Sertoli cell-conditioned media were examined by ELISA.

### 4.7. Extraction of Total RNA for Real-Time PCR Analysis

Extraction of RNA was performed using a Dynabeads RNA direct kit (Dynal Biotech, Oslo, Norway), as described [31]. Real-time quantitative PCR amplification of total cDNA (500 ng/sample) used specific primers of the different sequences: IL-34-Fw: 5′ TCACGTGGAAGCTGTGTTATCT, Rw: 5′ ACAGTACAGCAGTTCCATGACC, and GAPDH: Fw: 5′ ACCACAGTCCATGCCATCAC, Rw: 5′ CACCACCCTGTTGCTGTAGCC. The reactions were conducted following the protocol for the Absolute qPCR SYBR Green mix (ABgene House, Epsom, UK) containing modified Tbr DNA polymerase, SYBR Green, optimized PCR buffer, 5 mM MgCl2, dNTP mix, and dUTP. The PCR reaction was performed as described by [29]. The results were expressed as fold of increase related to the b-actin of the same examined sample.

### 4.8. Isolation of Tubular/Spermatogonial Cells

The tubular cells were isolated from the testes of 7-day-old ICR mice as described by Abu Elhija et al., 2012 [26]. The cells were suspended in StemPro-34 and were counted under phase-contrast microscopy in a Neubauer counting chamber (improved Double Neubauer Ruling, Saarlouis, Saaringia, Germany)

### 4.9. Culture of Isolated Spermatogonial Cells In Vitro in Methylcellulose Culture System (MCS)

The isolated tubular cells were cultured (2 × 10^5^ cells/well/500 µL) in methylcellulose (R&D Systems, Minneapolis, MN, USA) (42%) as a three-dimensional (3D) culture and in the presence of FCS (25%) and RPMI (33%) according to our previous studies [26,27,33]. Those cultures were growing in the absence (CT) or presence of recombinant IL-34 (recombinant mouse IL34, BioLegend; final dilution 1–10,000 pg/mL). The cells were incubated for four weeks in a CO_2_ incubator at 37 °C. Every 10–14 days, 50 µL/well of fresh media containing the relevant concentration of IL-34 were added to the cell cultures. At the end of the incubation period in MCS, 0.5 mL of PBS was added to the culture wells that contained 0.5 mL MC, mixed by pipetting, and collected from the suspension in 15-mL tubes. The tubes were centrifuged at 1600 rpm for 10 min. Most of the volume was removed and the remainder of around 100 µL was collected from the bottom of the tubes. This volume, which contained the cells, was smeared on a slide for histological examination and/or collected and kept at −70 °C in a lysis solution to be used for RNA extraction.

### 4.10. Data Handling and Statistical Evaluation

In each single experiment we made at least three repeats (three wells) for each treatment. Also, each experiment was repeated at least three times. The plotted data are the means calculated from a minimum of three independent experiments. The standard error of the mean (SEM) represents the variability between independent experiments. The Student’s *t*-test was used for the estimation of statistical significance and *p*-values below 0.05 were considered significant.
